# Clinical Application of Development of Nonantibiotic Macrolides That Correct Inflammation-Driven Immune Dysfunction in Inflammatory Skin Diseases

**DOI:** 10.1155/2012/563709

**Published:** 2012-11-01

**Authors:** Carmen Rodriguez-Cerdeira, Elena Sanchez-Blanco, Alberto Molares-Vila

**Affiliations:** ^1^Dermatology Department, CHUVI and University of Vigo, Vigo, Spain; ^2^University of Vigo, Vigo, Spain; ^3^Analytical Chemistry Department, University of Vigo, Vigo, Spain

## Abstract

*Background*. Inflammation-driven immune dysfunction supports the development of several chronic human disorders including skin diseases. Nonantibiotic macrolides have anti-inflammatory and/or immunomodulatory activity that suggests the exploitation of these in the treatment of skin diseases characterized by inflammatory disorders. *Materials and Methods*. We performed an extensive review of the nonantibiotic macrolide literature published between 2005 and 2012, including cross-references of any retrieved articles. We also included some data from our own experience. *Results*. Calcineurin antagonists such as tacrolimus and ascomycins (e.g., pimecrolimus) act by inhibiting the activation of the nuclear factor for activated T cells (NFAT). There are new applications for these macrolides that have been available for several years and have been applied to skin and hair disorders such as atopic dermatitis, oral lichen planus, vitiligo, chronic autoimmune urticaria, rosacea, alopecia areata, pyoderma gangrenosum, Behcet's disease, neutrophilic dermatosis, and lupus erythematosus. We also reviewed new macrolides, like rapamycin, everolimus, and temsirolimus. In addition to the literature review, we report a novel class of nonantibiotic 14-member macrocycle with anti-inflammatory and immunomodulatory effects. *Conclusions*. This paper summarizes the most important clinical studies and case reports dealing with the potential benefits of nonantibiotic macrolides which have opened new avenues in the development of anti-inflammatory strategies in the treatment of cutaneous disorders.

## 1. Introduction

The term “macrolide” encompasses a diverse family of unrelated compounds with large macrolactam rings. The activity of these compounds stems from the presence of a macrolide ring. Macrolide rings are comprised of a large macrocycle lactone ring to which one or more deoxy sugars, usually cladinose and desosamine, may be attached. In addition to their antibacterial activity, macrolides have diverse biological effects, including modulation of inflammatory and immune responses without affecting homeostatic immunity [1, 2].

 Macrolides are effective antibiotics that have immunomodulatory effects and inhibit the production of many proinflammatory cytokines such as interleukin 6 (IL-6), IL-8, and tumor necrosis factor alpha (TNF*α*). Macrolides are used in inflammatory skin and hair disorders. Many studies have been performed to assess their effectiveness in the treatment of rosacea, psoriasis, pityriasis rosea, alopecia areata, bullous pemphigoid, and pityriasis lichenoides [3].

However, new strategies for the treatment of cutaneous pathologies are directed towards the development of new nonantibiotic macrolides with anti-inflammatory, antiproliferative, and antiangiogenesis properties. The most known and used are inhibitors of the phosphatase calcineurin (pimecrolimus and tacrolimus), which under normal circumstances induce the transcription of IL-2. In addition, these drugs inhibit lymphokine production and interleukin release, which lead to a reduced function of effector T-cells [4]. 

Nowadays, novel chemical structures with improved therapeutic anticancer and anti-inflammatory properties by affecting skin disease targets have arose from mammalian rapamycin inhibitors. These agents inhibit the response to IL-2 and thus block the activation of T and B lymphocytes [5, 6].

Recently, new synthetic derivatives of the macrolide azithromycin, namely, CSY0073, (8R,9S)-8,9-dihydro-6,9-anhydropseudoerythromycin A (EM900), and (8R,9S)-4′′,13-O-diacetyl-8,9-dihydro-6,9-epoxy-8,9-anhydropseudoerythromycin A (EM911) having potent anti-inflammatory properties have been developed [6]. Currently, ridaforolimus has been developed but has only been used *in vitro* thus far. More studies are required to uncover the possible applications of these promising molecules although one of the first possible applications of these compounds is as an antitumor agent [7]. 

In this paper, we review the clinical use of nonantibiotic macrolides that have become available clinically for chronic inflammatory skin diseases with immune dysfunction.

## 2. Methodology

We searched the Cochrane Central Register of Controlled Trials (Central), Med-Line (PubMed), and Embase (2005 to January 2012). We also examined references from selected articles. We included case series with 5 or more patients, cohort trials, and randomised controlled trials. Search terms used were: “tacrolimus”, “pimecrolimus”, “calcineurin inhibitors”, “new macrolides”, “rapamycin”, and so forth and “atopic dermatitis”, “psoriasis”, and other common dermatitises that have been treated using macrolides. We also include some data from our own experience.

## 3. Results and Discussion

We have divided the paper into 2 sections.

### 3.1. Innovative Use of Calcineurin Inhibitors

#### 3.1.1. Pimecrolimus

**Figure 1:**
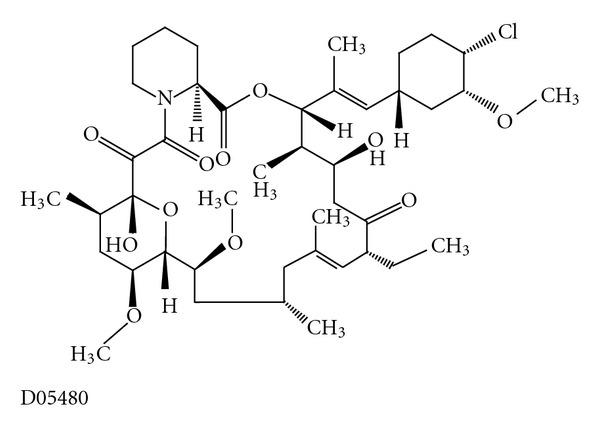
Molecular structure of pimecrolimus extracted from Kyoto Encyclopedia of Genes and Genomes (KEGG) database [8].

**Figure 2:**
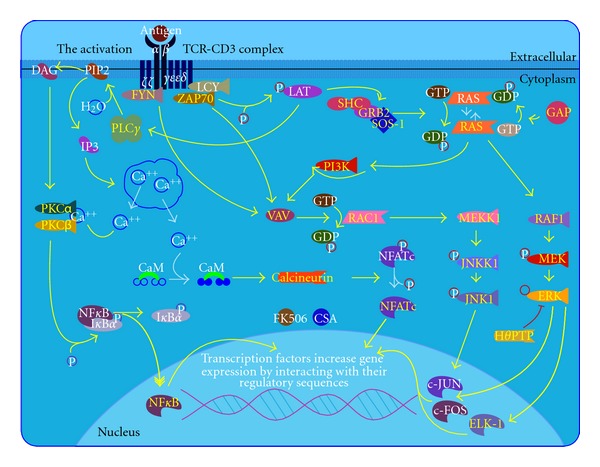
T cell receptor signaling pathway extracted from BioCarta database [9].

**Figure 3:**
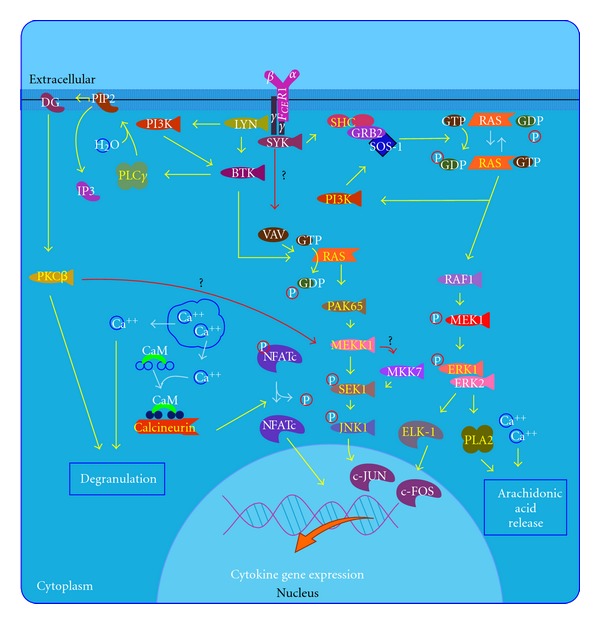
Fc epsilon receptor I signaling in mast cell pathway extracted from BioCarta database [9].

Pimecrolimus (SDZ ASM 981, Novartis) is one of the new classes of novel ascomycin immunomodulating macrolactams and was developed for the treatment of inflammatory skin diseases (Figure 1) [8]. Ascomycin, first isolated as a fermentation product of *Streptomyces hygroscopicus* var. ascomycetes, in the early 1960s, was initially researched for its antifungal properties. However, more than 20 years later, ascomycin was investigated for its structural and immunomodulatory properties. Pimecrolimus is a colourless, solid compound with a molecular weight of 810.48 Daltons. Interest in pimecrolimus has been intense because it has significant anti-inflammatory and immunomodulatory activity and because it has low potential for systemic immunosuppression [4]. The mechanism of action of pimecrolimus involves the blockage of T cell activation. Ascomycin macrolactams are immunophilin ligands that bind to a specific cytosolic receptor. Pimecrolimus binds to FKBP-12 and immunophilin macrophilin-12, also known as FK506 binding protein. Like tacrolimus and cyclosporin A, pimecrolimus acts by binding to macrophilin-12. The pimecrolimus-macrophilin complex then binds to the cytosolic enzyme calcineurin phosphatase. Calcineurin is a Ca^2+^/calmodulin-dependent protein phosphatase that regulates the translocation of the cytosolic components of NFATs. NFATs, in turn, regulate the promoter activities of several mediators during mRNA transcription. By inhibiting the action of calcineurin, the pimecrolimus-macrophilin complex prevents the dephosphorylation of the cytoplasmic component of NFATs. NFATs regulate the mRNA transcription of a number of inflammatory cytokines. Therefore, pimecrolimus blocks the transcription of these cytokines, especially T-helper Th1 (IL-2-, IFN-*γ*-) and Th2 (IL-4-, IL-10-) type cytokines (Figure 2) [8]. Pimecrolimus decreases the production of other cytokines, including interleukins IL-5, IL-10, and TNF*α*, in a dose-dependent manner [4]. Pimecrolimus also targets mast cells, which play an important role in anti-inflammatory activities. Pimecrolimus inhibits not only the transcription and synthesis of cytokines from mast cells, but also inhibits the release of the preformed mediators serotonin and  *β*-hexosaminidase. Additionally, pimecrolimus inhibits Fc Epsilon RI-mediated degranulation and secretion (Figure 3) [9]. It is important to note that all of these inhibitory processes occur only when pimecrolimus is bound to macrophilin-12. In a study of murine mast cell line CPII, pimecrolimus did not inhibit the transcription of a reporter gene that was under the control of human TNF*α*  promoter in the murine dendritic cell line and had no effect on IL-8 release from keratinocytes, fibroblasts, and endothelial cells. This is an indication of the specificity of the pharmacologic activity of pimecrolimus.

Atopic dermatitis (AD) is a pruritic disease of unknown origin that usually develops in early infancy (an adult-onset variant is recognized); it is characterized by pruritus, eczematous lesions, xerosis (dry skin), and lichenification (thickening of the skin and an increase in skin markings). AD may be associated with other atopic (immunoglobulin-E-(IgE-) associated) diseases (e.g., acute allergic reaction to foods, asthma, urticaria, and allergic rhinitis) [10]. Treatment of AD is one of the best known applications for pimecrolimus. Pimecrolimus inhibited cytokines, IL-2 and interferon gamma IFN*γ*, and Th2-type cytokines, IL-4 and IL-10. In addition, pimecrolimus prevents the release of inflammatory cytokines and mediators from mast cells *in vitro* after stimulation by antigen/IgE. References are still emerging in the literature for AD treatment with these drugs. Pimecrolimus cream 1% is a good option for treatment of mild to moderate AD in adults and children aged ≥2 years [4, 10]. No novel systemic applications have appeared since 2005. In 2005, however, there was a study of oral pimecrolimus for use in the treatment of moderate to severe AD. This study demonstrated the efficacy and short-term safety of oral pimecrolimus in adults in a double-blind study with a 12-week treatment and 12-week post-treatment phase. Longer-term studies in larger cohorts are now required [11].

Psoriasis is considered a chronic skin condition. However, its exact cause remains unknown. Psoriasis may develop because of a combination of factors, including genetic predisposition and environmental factors. Psoriasis may be commonly observed among members of the same family. The immune system is thought to play a major role in the development of this condition. Psoriasis has a variable course, which periodically improves and worsens. Many people note a worsening of their symptoms in the colder winter months. Psoriasis produces red, dry plaques of thickened skin. The dry flakes and skin scales are thought to result from the rapid proliferation of skin cells that is triggered by abnormal lymphocytes in the blood. Psoriasis commonly affects the skin of the elbows, knees, and scalp [12]. Another important application for pimecrolimus is psoriasis treatment, where it acts through blockage of T-cell activation and signal transduction pathways in T cells and through inhibition of the synthesis of inflammatory cytokines, which play a key role in the pathogenesis of psoriasis [13]. Oral pimecrolimus was tested in healthy adult outpatients with moderate to severe chronic plaque-type psoriasis (*n* = 143) who received either an oral placebo or pimecrolimus for 12 weeks. Oral pimecrolimus was well tolerated and produced a dose-dependent reduction in psoriasis severity. Doses of 20 mg and 30 mg b.d. were the most effective [14, 15].

**Figure 4:**
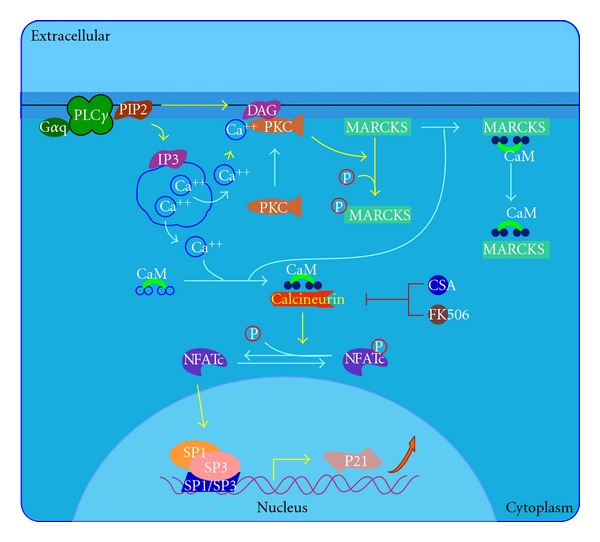
Effects of calcineurin in keratinocyte differentiation pathway extracted from BioCarta database [9].

Oral lichen planus (OLP) is an inflammatory condition that affects the mucous membranes of the mouth. OLP may appear as white lacy patches, red swollen tissues, or open sores. These lesions may cause burning, pain, or other discomfort. OLP is a T-cell-mediated chronic inflammatory oral mucosal disease of unknown cause, and lesions contain few B cells or plasma cells and minimal deposits of immunoglobulin or complement. Therefore, OLP is ideal for studying human T-cell-mediated inflammation and autoimmunity. Antigen-specific mechanisms in OLP include antigen presentation by basal keratinocytes and antigen-specific lysis of keratinocytes by CD8+ cytotoxic T cells. Nonspecific mechanisms include mast cell degranulation and matrix metalloproteinase activation in OLP lesions. A combination of these mechanisms may cause T cell accumulation in the superficial lamina propria, basement membrane disruption, intraepithelial T cell migration, and apoptosis of keratinocytes in OLP (Figure 4) [9, 16]. Pimecrolimus, as described above, inhibits dephosphorylation of nuclear factor of activated T cells by calcineurin, thus, reducing T-cell cytokine production and inhibiting T-cell activation. Pimecrolimus significantly reduces the symptoms of OLP [17, 18].

Vitiligo is a common depigmenting disorder affecting about 1-2% of the world population. Approximately half of the affected individuals develop the disease before adulthood. Etiologic hypotheses for vitiligo include biochemical, neural, and autoimmune mechanisms. The most compelling of these suggests a combination of genetic and immunologic factors that results in autoimmune melanocyte destruction. Pimecrolimus have comparable efficacy and a better safety profile compared with topical corticosteroids. It was effective in their treatment better than topic corticoids [19, 20].

Patients in whom the cause of urticaria is unknown are said to have chronic idiopathic urticaria; however, findings suggest that in 25–45% of patients, chronic idiopathic urticaria is not idiopathic but is an autoimmune disease termed as chronic autoimmune urticaria [21]. Chronic autoimmune urticaria is dependent not only on the cross-linking of IgE receptors (by anti-Fc Epsilon RIa or anti-IgE), but also on the activation of complement. Cross-linking of IgE receptors leads to histamine release via a calcineurin-dependent signal transduction pathway, whereas complement C5a receptors act through G-proteins. Histamine release by patient sera or isolated IgG can be inhibited by ascomycin but not the C5a. The failure of pimecrolimus to satisfactorily treat chronic autoimmune urticaria may at least in partly result from this [22]. 

Rosacea is a common cutaneous disorder, which occurs most frequently in light-skinned middle-aged individuals. Cutaneous signs are flushing, erythema, telangiectasia, and papules and pustules. An important reference we found to the use of pimecrolimus for the treatment of rosacea was a study “by Kim” in 26 patients with mild to moderate inflammatory rosacea [23].

Alopecia areata (AA) is an autoimmune disease of the hair follicle caused by a T-lymphocytic infiltrate, although its pathogenesis is not yet completely clear. AA results in hair loss and baldness, and may frequently remit and relapse. Histologically, the peribulbar infiltration consists mainly of activated CD4+ and CD8+ T-cells. Type 1 cytokines, including IL-2, IFN-c, and TNF*α*, mediate initiation of the immune response in AA. Pimecrolimus prevents calcineurin-mediated dephosphorylation of the NFATs, which inhibits the synthesis of Th1 and Th2 cytokines in T lymphocytes. Topical pimecrolimus treatment is as effective as topical corticosteroids for the treatment of AA and frontal fibrosing alopecia, and has fewer side effects than topical corticosteroids [24, 25].

Pyoderma gangrenosum (PG) is an uncommon ulcerative cutaneous condition of uncertain cause. PG is associated with systemic diseases in at least 50% of the patients. This condition is diagnosed by excluding other causes of similar-appearing cutaneous ulcerations, including infection, malignancy, vasculitis, collagen vascular diseases, diabetes, and trauma. Pathergy involves development of new ulcerations after trauma or injury to the skin in 30% of patients with existing PG. The pathogenesis of PG is not entirely understood, but defects in cell-mediated immunity, humeral immunity, neutrophil chemotaxis, and monocyte phagocytosis along with diminished lymphokine production have been observed in patients with PG [26]. Positive clinical results from treatment of PG with pimecrolimus and tacrolimus are probably due, in part, to a decrease in the release of TFN*α*. TFN*α*  release is considered to be very important in the development of the neutrophilic dermatoses. Pimecrolimus does not affect the differentiation, maturation, and functions of Langerhans cells and does not induce their apoptosis [27]. 

Discoid lupus erythematosus (DLE) is a chronic skin condition of sores with inflammation and scarring on the face, ears, and scalp, and at times, on other areas of the body. These lesions develop as a red inflamed patch with a scaling and crusty appearance. Localized DLE typically manifests as skin lesions localized above the neck and mainly involves sites such as the scalp, bridge of nose, cheeks, lower lip, and ears [28]. Lesions have elevated levels of IL-2, IFN*γ*, and TNF*α*  mRNA, as compared to normal skin. Elevated type I IFN (IFN-*α*/*β*) has also been found in these skin lesions. Type 1 IFN is correlated with Th1-associated inflammation. In addition, unlike cyclosporine and tacrolimus, the action of pimecrolimus is more selective for T-cells and mast cells, thus reducing the likelihood of systemic immunosuppression [29].

Behçet's disease (BD) was named in 1937 after the Turkish dermatologist Hulusi Behçet who first described the triple-symptom complex of recurrent oral aphthous ulcers, genital ulcers, and uveitis. Painful genital ulcerations usually develop around the anus, vulva, or scrotum and cause scarring in 75% of the patients. The cause is not well defined, but it is primarily characterized by autoinflammation of the blood vessels. The primary mechanism of the damage is an overactive immune system that seems to target the patient's own body. The primary cause is not well known. In fact, as of now, no one knows why the immune system starts to behave this way in Behçet's disease. There does however seem to be a genetic component involved, as first degree relatives of the affected patients are often affected in more than expected proportion for the general population [30]. Pimecrolimus is safe and effective for the treatment of BD genital ulcers and accelerates the healing process [31].

Graft-versus-host disease (GVHD) is a common complication of an allogeneic tissue transplant. GVHD is commonly associated with stem cell or bone marrow transplant, but the term also applies to other forms of tissue graft. Immune cells (white blood cells) in the tissue (the graft) recognize the recipient (the host) as “foreign.” Subsequently, the transplanted immune cells attack the cells of the host's body. GVHD can also occur after a blood transfusion if irradiated blood products are not used. In the classical sense, acute GVHD is characterized by selective damage to the liver, skin (rash), mucosa, and the gastrointestinal tract. New research indicates that target organs of GVHD other than those mentioned above include the immune system (the hematopoietic system, e.g., the bone marrow and the thymus) itself, and the lungs in the form of idiopathic pneumonitis. Further, chronic GVHD involves the above organs but can also cause damage to the connective tissue and exocrine glands over a long term. T cells present in the graft, either as contaminants or intentionally introduced into the host, attack the tissues of the transplant recipient after perceiving host tissues as antigenically foreign. The T cells produce an excess of cytokines, including TNF-*α*  and interferon-gamma (IFN*γ*). A wide range of host antigens can initiate GVHD, such as the human leukocyte antigens (HLAs). The only study in which the treatment of GVHD was reported was that by Schmook. Further research is required to address this issue [32].

#### 3.1.2. Tacrolimus

**Figure 5:**
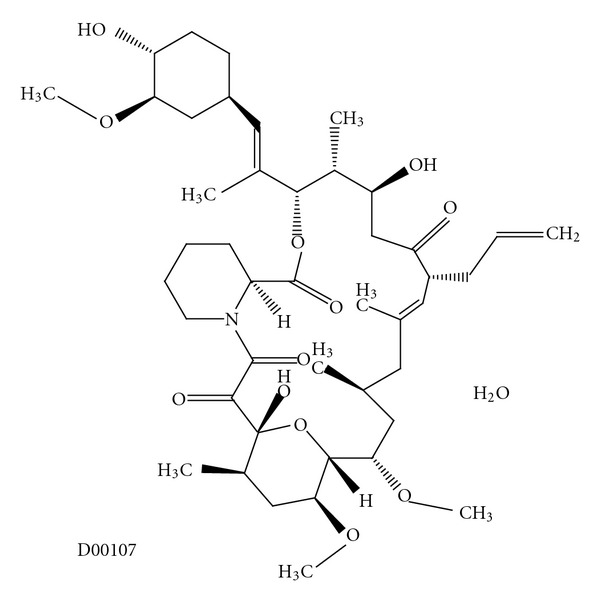
Molecular structure of tacrolimus extracted from KEGG database [9].

**Figure 6:**
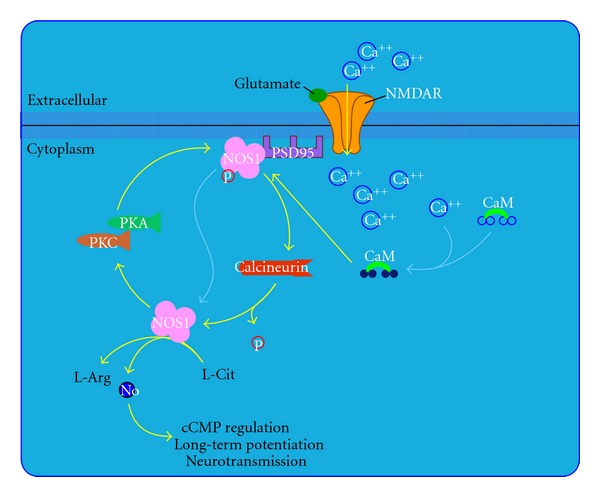
Nitric oxide signaling pathway extracted from BioCarta database [9].

**Figure 7:**
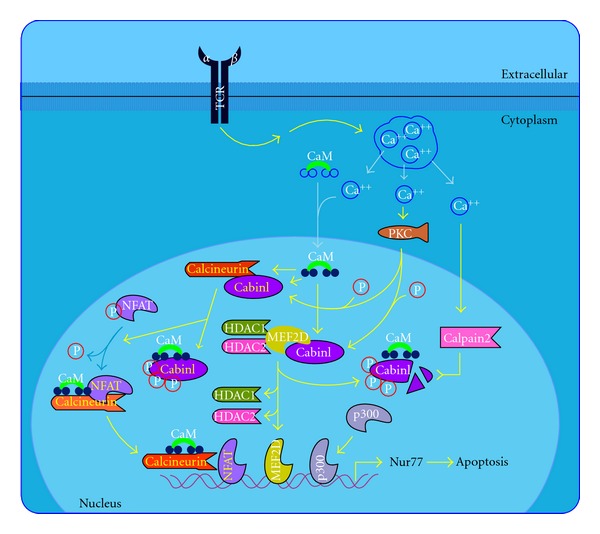
T cell apoptosis pathway extracted from BioCarta database [9].

Tacrolimus (Figure 5) [9] was first isolated in 1984 from a Japanese soil fungus. Tacrolimus is structurally dissimilar to cyclosporine, but has similar immunosuppressive properties. The macrolide antibiotic tacrolimus (FK 506) was discovered as a naturally occurring metabolite of the fungus *Streptomyces tsukubaensis*. Tacrolimus is a “prodrug” that becomes active after forming complexes with intracytoplasmic proteins called immunophilins. Once activated, tacrolimus binds to FKBP. At least 4 FKBP are described: 12, 13, 25, and 59. The main effect of tacrolimus appears to result from the inhibition of T-cell function. Following the binding of an antigen-presenting cell to a T cell via the T cell receptor, intracytoplasmic levels of calcium rise, leading to calmodulin activation of the phosphorylase enzyme, calcineurin phosphatase. Calcineurin phosphatase is the main target of this drug. The activation of calcineurin phosphatase leads to the dephosphorylation of a cytoplasmic protein-NFAT. Once dephosphorylated, NFAT translocates into the nucleus where it combines with a nuclear subunit (NFATn). The resulting nuclear complex binds to the promoter units of several genes. The binding of NFATn enables transcription of proinflammatory cytokines, including IL-2, IL-4, IFN*γ*, and TGF-*β*  and upregulation of receptors, such as IL-2R (CD25). Transcription of these cytokines initiates T-cell activation (Figure 2) [9, 33]. Activated tacrolimus inhibits the action of calcineurin, thus preventing the dephosphorylation of nuclear factors and blocking this path to gene transcription. In stimulated T cells, tacrolimus inhibits activation principally by suppressing IL-2 production and IL-2R expression. Inhibition of IL-2 production blocks the activation of T-helper cells, T-regulatory cells (autocrine loop), natural killer cells, and monocytes. In addition to inhibiting IL-2 transcription, other calcium-dependent events, including nitric oxide synthase activation (Figure 6) [9], cell degranulation, and apoptosis (Figure 7) [9] are also inhibited. In stimulated mast cells, tacrolimus decreases histamine release, impairs Langerhans' cell function, and downregulates high-affinity IgE receptors. It also decreases the production of chemotactic protein-1 and IL-8 in monocytes and affects other cell types, including neutrophils, eosinophils, and endothelial cells. Inhibition of calcineurin interferes with superantigen stimulation of T cells and may decrease the production of vascular endothelial growth factor. Tacrolimus also inhibits the function of B cells and the production of other cytokines such as IL-3, IL-4, IL-5, IFN*γ*, TNF*α*, and granulocyte-macrophage colony stimulating factor (GM-CSF) [34].

When used to treat AD, tacrolimus inhibits the T lymphocytes, which release the cytokines that trigger the inflammation underlying AD. Tacrolimus also affects other cells including Langerhans and mast cells. By downregulating T cells, the symptoms of AD begin to fade within a few days of applying a topical ointment that contains tacrolimus. Such ointments penetrate the skin sufficiently to allow local immunomodulation. However, the skin does not act as a reservoir for this drug, as discussed by Kim and Kono [35]. Oral tacrolimus is an additional therapeutic option for management of severe and extensive AD [36].

Tacrolimus inhibits the production of many proinflammatory cytokines, such as IL-6, IL-8, and TNF*α*, perhaps by suppressing the transcription factors NF-*κ*B or activator protein-1. It also reduces neutrophil activity. Studies of topical tacrolimus as a treatment for psoriasis have yielded disappointing results. However, topical tacrolimus that was applied under occlusion to descaled psoriatic plaques is an effective treatment. There is good evidence that topical tacrolimus is a highly effective treatment for psoriasis of the face and flexures [37–39].

In our clinical practice, treatment with 0.15 mg/kg b.d. oral tacrolimus for 1 week resulted in a marked reduction in the erythema and scaling of severe psoriasis patients. Complete remission occurred after 4 weeks of treatment. Administration of tacrolimus at a dose of 0.3 mg/kg per day to 7 patients with recalcitrant psoriasis resulted in remission with minor metabolic effects, including minimal elevation of urea, creatinine, and glucose in the blood [40].

More recently, tacrolimus has been used to treat genital lichen sclerosus, a condition in which patches of the skin become thin and wrinkled. Thus, the skin tears easily, and bright red or purple bruises are common. Sometimes, the skin becomes scarred. Tacrolimus blocks the proliferation of T lymphocytes and the release of inflammatory cytokines from these cells. The skin on the patches becomes thin and crinkled. Then the skin tears easily, and bright red or purple bruises are common [41]. Sometimes, the skin becomes scarred. If the disease is a mild case, there may be no symptoms. Tacrolimus ointment 0.1% may also be effective and well tolerated for the treatment of anogenital lichen sclerosus, both in adults and in prepubertal girls. Active lesions cleared in 43% of patients after 24 weeks of treatment. Partial resolution was reached in 34% of patients [42]. Recent reports describe the use of 0.1% tacrolimus in a topical formulation for the management of OLP [43]. Therefore, there is a need for more effective and safer therapies for symptomatic OLP. The activation of IL-2 production occurs after antigen, with a major histocompatibility complex type II antigen, is presented to the T-cell receptor-CD3 complex. Antigen presentation results in the release of calmodulin, which binds and activates the protein calcineurin that is involved in the dephosphorylation of NFAT. The activated NFAT induces the transcription of the IL-2 gene. Tacrolimus and the intracellular immunophilin protein known as the FK-binding protein form a complex that binds to and inactivates the protein calcineurin. As a result, the T-cell receptor-mediated induction of IL-2 production is inhibited, resulting in suppression of T-cell-dependent immune functions [43, 44].

Contact dermatitis is a condition in which the skin becomes red, sore, or inflamed after direct contact with a substance. There are 2 types of contact dermatitis: irritant and allergic. Treatment of contact dermatitis is often palliative and directed against cutaneous inflammation itself. Tacrolimus has good anti-inflammatory effects and penetrates well through inflamed skin. In a human study, topical tacrolimus (at concentrations of 0%, 0.01%, 0.1%, and 1%) in ethanol were applied to the skin of 5 volunteers and left for 48 hours. 1-Chloro-2,4-dinitrobenzene (DNCB) was then applied to the skin. Biopsies of the test patches showed no inflammation on the DNCB-challenged skin sites that were pretreated with FK 506, while there was intense dermatitis ethanol-only. The ability to suppress reactions in previously sensitized patients is important because contact dermatitis patients do not present until after primary sensitization. The ability to treat such sensitized individuals is crucial because many antigens, such as nickel, are ubiquitous and complete avoidance is often impossible. Topical tacrolimus also suppresses irritant reactions in animal models, suggesting that topical tacrolimus may also be useful for primary irritant contact dermatitis. This may be applicable to the treatment of chronic hand dermatitis and occupational irritant dermatitis (in which allergic contact often coexists) [45].

The efficacy and safety of 0.1% tacrolimus ointment in vitiligo were compared favourably to that of 0.05% fluticasone propionate cream for the treatment of segmental vitiligo in a randomized controlled trial [46].

Even diseases that are not considered to be classic T-cell-mediated inflammatory processes have been considered as targets for tacrolimus therapy. Goldman noted that the anti-inflammatory properties of topical tacrolimus that unlike steroids, tacrolimus may not have intrinsic rosacea-promoting properties. He treated the patients who had steroid-induced rosacea and were previously unable to taper off and discontinue the use of steroid therapy. The eruptions were controlled in all 3 patients, and they were able to successfully taper off tacrolimus therapy and switch to a long-term regimen of topical antibiotics [47, 48].

While AA is another candidate disease for tacrolimus therapy, some authors have expressed reservations regarding its use for this purpose, as AA generally responds poorly to treatment. Thiers published a report of the failure of 0.3% tacrolimus ointment to treat AA in a 9-year-old [49]. We found no other descriptions of AA tacrolimus therapy published after 2000. Steroid intralesionals in combination with topically applied tacrolimus yield better results than topical tacrolimus alone [50, 51]. In 50–75% of patients, PG is associated with inflammatory bowel disease, rheumatoid arthritis, chronic autoimmune hepatitis, or haematological solid tumours. Some reports have indicated that topical tacrolimus is an effective treatment for PG. Immunosuppressive agents have also been used for the management of PG [50–53].

Tacrolimus ointment (0.1%) was applied to DLE lesions twice daily and the erythematous plaques readily diminished after 4–8 weeks. Adverse effects, such as burning sensation or irritations, were not observed. Cutaneous LE is a broad term, which includes a variety of lesions that may appear in the absence of the systemic manifestations of systemic lupus erythematosus [52, 53]. In an open-label study of tacrolimus (0.1 mg/kg) administered for 1 year with dosage adjustment showed that serum C3 level, and anti-ds DNA antibody titre improved with tacrolimus treatment. The mean titre of anti-ds DNA antibodies provides a representative indicator of immunological parameters reflecting disease activity. Therefore, a T cell blockade is considered a reasonable therapeutic target for cutaneous and systemic LE [54, 55]. Dosages differ between reports (1.5–6 mg/day). Tacrolimus can therefore be considered both effective and safe for treating mild manifestations of LE, including skin dermatosis, in systemic LE patients. However, for severe active conditions, its efficacy is limited at current dose settings and usage [56].

Crohn's disease (CD), also known as regional enteritis, is a type of inflammatory bowel disease that may affect any part of the gastrointestinal tract from the mouth to anus and causes a wide variety of symptoms. CD is caused by interactions between environmental, immunological, and bacterial factors in genetically susceptible individuals. This results in a chronic inflammatory disorder, in which the immune system of the body attacks the gastrointestinal tract possibly directed at microbial antigens. In addition, CD may involve the skin, blood, and endocrine system. One type of skin manifestation, erythema nodosum, presents as red nodules usually appearing on the shins. Erythema nodosum is due to inflammation of the underlying subcutaneous tissue and is characterized by septal panniculitis. Another skin lesion, pyoderma gangrenosum, is typically a painful ulcerating nodule. A new view is that CD results from an impaired innate immunity, in that impaired cytokine secretion by macrophages contributes to impaired innate immunity and leads to a sustained microbial-induced inflammatory response in the colon, where the bacterial load is high [57]. Despite the poor quality of the majority of trials examining the role of tacrolimus in CD, there is some evidence suggesting that tacrolimus may be of some benefit in this disease. Although systemic immunosuppressants are generally believed to increase the rate of cancer development, one study has shown that in female CD-1 mice there was a dose-related inhibition of 7,12-dimethylbenz[a]anthracene-(DMBA-) initiated and 12-tetradecanoylphorbol-13-acetate-(TPA-) promoted skin papillomas when 0.1 *μ*mol tacrolimus was applied topically. The application of this formulation to mouse skin almost completely inhibited tumour formation. This antineoplastic effect may be unrelated to the suppression of T-cell functions and might occur after endogenous protein phosphorylation by TPA. This study was contradicted by a later study of the occurrence of de novo neoplasms in organ transplant recipients. This later study indicated that tacrolimus is as an inducer of skin cancer [58].

Cutaneous T cell lymphoma (CTCL) is a class of non-Hodgkin's lymphoma, which is a type of cancer of the immune system. The malignant T cells in the body initially migrate to the skin, which result in the development of various lesions. These lesions change shape as the disease progresses, typically beginning as what appears to be a rash, which can be very itchy, and eventually forming plaques and tumors before metastasizing to other parts of the body. CTCLs are a heterogeneous group of lymphoproliferative disorders caused by clonally derived skin-invasive T cells. Few studies have reported the efficacy of topical tacrolimus for the treatment of CTCLs [59–61].

Topical application of 0.3% tacrolimus in isotonic solution or cream is a promising treatment modality for pathology ocular in BD [62]. Pulmonary and intestinal lesions evanesced and skin lesions improved after the oral administration of FK506 at a dose of 0.1-0.2 mg/kg for 8 weeks [63, 64]. 

In a case of refractory GVHD, the patient responded to a combination of oral tacrolimus, psoralen, and UV-A therapy. This suggests that systemic tacrolimus may benefit recipients of solid organ or bone marrow transplants with GVHD that is refractory to cyclosporine, high-dose systemic steroids, and antithymocyte globulin [65]. Tacrolimus is effective in the prevention of acute GVHD. The initial intravenous FK506 dose of 0.04 mg/kg per day and should be maintained for 7 days post-transplant. After day 7, intravenous FK506 doses should be decreased if serum creatinine is elevated to approximately 0.03 mg/kg per day [66]. Sabry et al. suggested that tacrolimus and mycophenolate mofetil is a good option for prophylaxis in HLA-matched nonmyeloablative transplants [67].

 Sarcoidosis is a systemic inflammatory disease that can affect any organ. Sarcoidosis involves the skin in about 25% of patients. The most common lesions are erythema nodosum, plaques, maculopapular eruptions, and subcutaneous nodules. The exact cause of sarcoidosis is unknown. The current working hypothesis is that in genetically susceptible individuals, sarcoidosis is caused through an alteration in immune response after exposure to environmental, occupational, or infectious agents [68]. Granulomatous inflammation is characterized primarily by accumulation of monocytes, macrophages, and activated T lymphocytes with increased production of key inflammatory mediators, TNF-*α*, IFN*γ*, and IL-12, characteristic of a Th1-polarized response (T-helper lymphocyte-1 response). Sarcoidosis has contrasting effects on inflammatory processes; it is characterized by increased macrophage and CD4 helper T-cell activation, which results in accelerated inflammation; however, immune response to antigen challenges such as tuberculin is suppressed. Regulatory T lymphocytes in the periphery of sarcoid granulomas appear to suppress IL-2 secretion, which is hypothesized to cause a state of anergy, by preventing antigen-specific memory responses. Topical tacrolimus has proved effective for the treatment of cutaneous sarcoidosis [69].

In the recent years, tacrolimus has been used to suppress the inflammation associated with diverse autoimmune or granulomatous diseases. “As described by Alijotas-Reig,” 7 patients with severe and refractory late-onset inflammatory reactions, including large panniculitis, which complicate silicone gel injections were evaluated. After an average of 18 months after tacrolimus administration (in increasing doses, up to 0.08 to 0.1 mg/kg of body weight, 2 times per day), 5 patients experienced mild, sparse bouts of inflammatory processes, including nodules, plaques, and panniculitis. The symptoms were rapidly reversed, and 2 patients showed remission. No side effects related to tacrolimus were observed. The ability of silicone to initiate immunologic processes remains to be clarified. An exhaustive federally sponsored review failed to find evidence to support immunological effects [70].

Long-lasting implants of any type that interact with commensal or infectious microorganisms, trauma, or localized or generalized inflammatory processes could theoretically induce autoimmune disorders or granulomata. These events may occur because of epigenetic alterations in DNA expression in genetically susceptible hosts. An excellent candidate for pathologic mischief on the face is *Propionibacterium acnes* that under certain circumstances can act as an opportunistic pathogen, which stimulates the production of TNF-*α* and polysaccharides [71, 72].

### 3.2. Rapamycin and Future Directions in the Development of Mammalian Rapamycin Inhibitor Development

#### 3.2.1. Rapamycin

**Figure 8:**
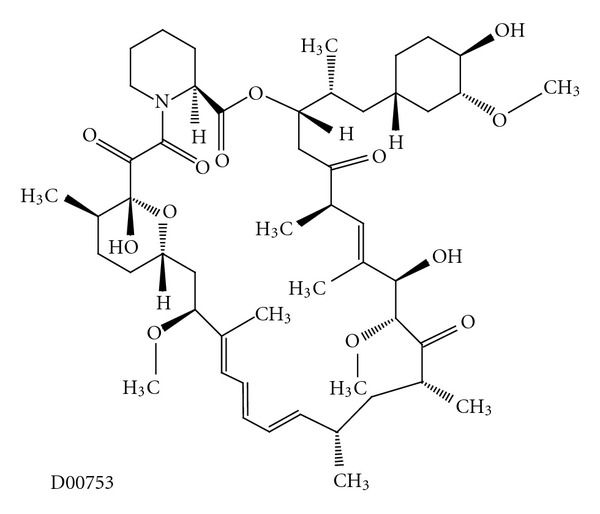
Molecular structure of sirolimus extracted from KEGG database [9].

**Figure 9:**
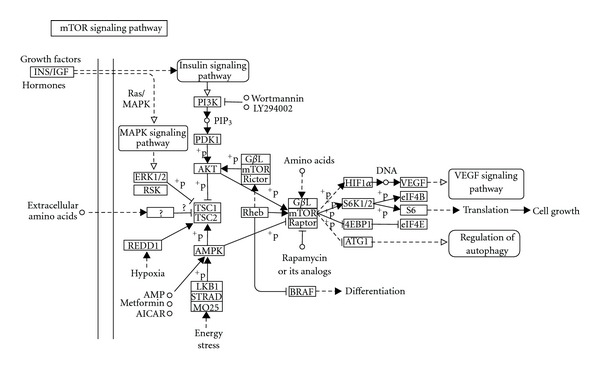
mTOR signaling pathway extracted from KEGG database [8].

Another widely used macrolide is rapamycin, also known as sirolimus (Figure 8) [9]. Rapamycin acts through the inhibition of mammalian target of rapamycin (mTOR), a molecule that is activated via phosphoinositide 3-kinase (PI3K) and controls downstream proteins involved in the cell cycle. After binding with tacrolimus binding protein (FK-BP) immunophilin, the rapamycin complex inhibits the stimulatory effect of mTOR on cell cycle protein translation, arresting the G_1_ to S transition (Figure 9) [8, 73]. This inhibitory effect can be partly explained because of a reduction of the phosphorylation of eIF-4e binding protein 1 (4E-BP1), a repressor of cap-mediated translation in mammalian cells [74]. Since 1999, rapamycin has been broadly used in human skin transplantation because it carries a low risk of renal dysfunction and reduces the risk of allograft rejection in comparison with other [75–77].

Rapamycin may potentially be used as an antiangiogenic agent to inhibit, for example, growth of pathological blood vessels in combination with laser treatment [78]. The application of a laser to an area of skin provokes the shutdown of major capillary vessels and results in the induction of a severely hypoxic microenvironment. This can cause overexpression of hypoxia-inducible factor-1 alpha (HIF1*α*) and promote the secretion of angiogenesis-stimulating factors like platelet-derived growth factor (PDGF) [79] and vascular endothelial growth factor (VEGF) [80]. Rapamycin may prevent vascular reperfusion by acting as an inhibitor of this mTOR-HIF1*α*-VEGF pathway and through the inhibition of the PI3K-p70S6 kinase pathway in endothelial cells stimulated by VEGF [81, 82].

There are some side effects to take into account, such as mild cholangitis [75] and delays in wound closure [75, 83]. These side effects may result from the multiple effects rapamycin may exert upon mTOR inhibition in the epithelial and stromal tissues of the wound area. This includes the important role of mTOR in the wound healing process downstream from phosphatidylinositol 3 kinase (PI3K) and phosphatase and tensin homolog (PTEN) [84]. 

A randomized, double blind, left-right comparative, dose-ranging clinical trial was carried out to determine the efficacy and safety of rapamycin applied to skin for the treatment of psoriasis [85]. The trial showed that rapamycin was able to penetrate human skin and exerted beneficial effects. A few subjects, however, developed contact sensitization to rapamycin [85].

#### 3.2.2. Everolimus

**Figure 10:**
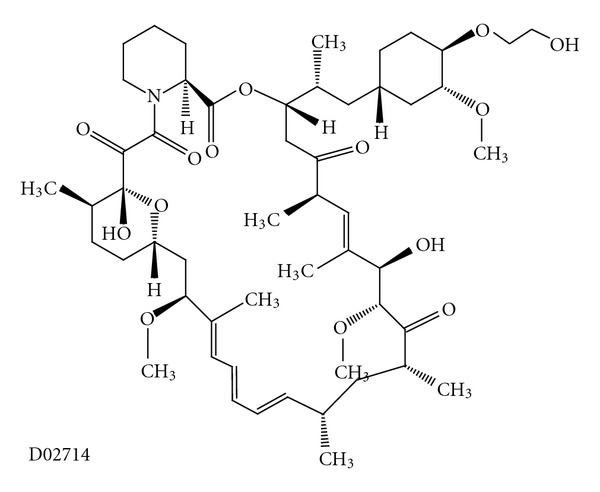
Molecular structure of everolimus extracted from KEGG database [9].

Everolimus (RAD001) is a rapamycin derivative (Figure 10) [9] with potent immunosuppressive effects, antiproliferative properties, and anticancer effects in many preclinical and clinical studies [86]. In addition, everolimus has shown *in vivo* antitumor activity with a significant cytostatic activity in a variety of preclinical models of haematological and solid tumours.

It has been reported that everolimus, while and effective treatment for psoriasis [87], became ineffective in 2 cases of severe atopic dermatitis when it was combined with prednisone or cyclosporine A [88]. More studies are needed to confirm this result.

#### 3.2.3. Temsirolimus

**Figure 11:**
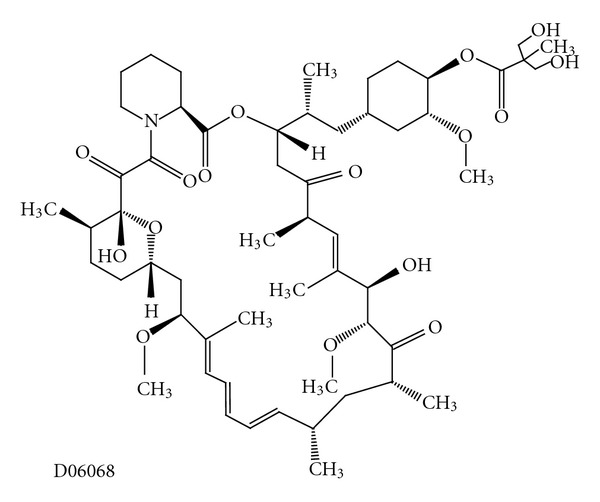
Molecular structure of temsirolimus extracted from KEGG database [9].

Temsirolimus (CCI-779, Torisel, Wyeth) is another rapamycin derivative (Figure 11) [9] and has properties that are similar to everolimus [86]. It has been used for the treatment of metastatic renal cell carcinoma and mantle cell lymphoma.

Rapamycin, everolimus, and temsirolimus all prevent tumour cell proliferation and angiogenesis through inhibition of the HIF1*α*/VEGF pathway [89–91].

#### 3.2.4. New Macrolides and Their Applications

A new synthetic azythromycin-derivative-macrolide, called CSY0073 [92], has anti-inflammatory and immune-modulatory effects, but no antibiotic effects. CSY0073 exerts counterregulatory activity on nuclear factor kappa B (NF-*κ*B), activator protein-1 (AP-1) and extracellular signal-regulated kinase 1/2 (ERK1/2) signalling. The anti-inflammatory activity of CSY0073 was demonstrated in rodent models of intestinal inflammation and hold potential as a treatment of inflammation-driven immune dysfunction. CSY0073 may reduce the colonic expression of cytokines involved in the development and maintenance of colon inflammation, such as tumour necrosis factor  *α*  (TNF*α*), interleukin 2 (IL-2), and interferon  *γ*  (IFN*γ*) [93]. In addition, CSY0073 effectively attenuated the immune response of mucosal macrophages. This is consistent with studies of other macrolides that indicate that these compounds penetrate the cell membrane of macrophages and accumulate in subcellular compartments [94]. CSY0073 is also being developed as a therapeutic drug for rheumatoid arthritis. Initial results indicate that treatment with CSY0073 attenuates the development of several signs of arthritis [92].

Recently, 2 new potential macrolides with anti-inflammatory and immunomodulatory characteristics were discovered. These compounds, (8R,9S)-8,9-dihydro-6,9-anhydropseudoerythromycin A (EM900) and (8R,9S)-4′′,13-O-diacetyl-8,9-dihydro-6,9-epoxy-8,9-anhydropseudoerythromycin A (EM911), are derivatives of erythromycin A [95]. EM900 and EM911 have so far only been used *in vitro*. More studies are needed to uncover the possible applications of these promising molecules.

**Figure 12:**
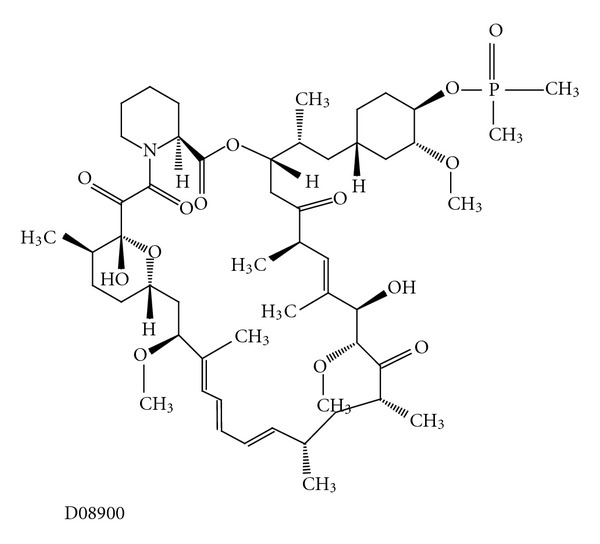
Molecular structure of ridaforolimus extracted from KEGG database [9].

Another newly developed molecule is ridaforolimus (also known as deforolimus, AP23473, MK-8669, Merck), a rapamycin analogue (Figure 12) [9], which has broad inhibitory effects on the cell growth, proliferation, division, metabolism, and angiogenesis of a broad panel of cell lines [96]. *In vitro* and *in vivo* studies show that ridaforolimus inhibits mTOR function in a selective and potent manner. Inhibitory effects on VEGF, endothelial cell growth (EGF), HIF-1*α*, and glucose metabolism were also observed. In particular, ridaforolimus was found to arrest cell growth without evidence of cell death or apoptosis, accumulating cells in the G_1_ phase of the cell cycle. This was due, in part, to a blockade of 4E-BP1/eIF4E signalling [97, 98]. One of the first possible applications for this compound is as an antitumour agent.

## 4. Conclusions

New uses are being developed for older macrolides, such as pimecrolimus and tacrolimus, due to their interesting anti-inflammatory properties. These drugs work through the inhibition of the calcineurin promotion of several cytokines, such as interleukins, interferons, and TNF*α*. This approach is opening a broad field of skin disease treatments that have minimal side effects (Table 1).

On the other hand, newer macrolides (rapamycin, everolimus, and temsirolimus) work through the downregulation of the mTOR pathway. The mTOR pathway controls downstream proteins that are involved in the cell cycle. These newer macrolides arrest the G_1_ to S transition, an important early event in the control of mammalian cell growth and proliferation. These macrolides also demonstrate antiproliferative, cytostatic, and antiangiogenic properties. There are many examples of successful applications for these compounds in cancer diseases and organ transplantation. These compounds have also been used in the treatment of skin diseases. There were a variety of responses to these compounds, and some of them were not at all positive. Further research in this field is required to determine potential applications for these macrolides.

## Figures and Tables

**Table 1:** Main advantages and drawbacks of different nonantibiotic macrolides in skin diseases.

Macrolide	Advantages	Drawbacks
Pimecrolimus	(i) Plays an important role in the anti-inflammatory activities. (ii) Applied for the treatment of atopic dermatitis (AD) [4, 9, 10]. (iii) Inhibits the synthesis of inflammatory cytokines in psoriasis [13, 14]. (iv) Produces a dose-dependent reduction in the severity of psoriasis [15]. (v) Significantly reduces the symptoms in oral lichen planus (OLP) [16, 17]. (vi) Applied for the treatment of rosacea [23]. (vii) Shows positive clinical results in pyoderma gangrenosum (PG) [27]. (viii) Acts more selectively on T cells and mast cells in lupus dermatosis and thus has a lower possibility of systemic immunosuppression [28, 29]. (ix) Is safe and efficient for the treatment of Behçet's disease genital ulcers, by accelerating the healing process [31].	(i) Tacrolimus is used more often for vitiligo [19, 20, 46]. (ii) Failure in treatment of chronic autoimmune urticaria [22]. (iii) Topical 1% is not a therapeutic option in alopecia areata (AA), especially for patients unresponsive to other treatments [24]. (iv) In PG, the pharmacologic activity is more selective than tacrolimus, and the rate of cutaneous permeation is 9 times lower than that of tacrolimus and, therefore, has a lower risk of systemic immune suppression [27].

Tacrolimus	(i) Oral formulation offers an additional therapeutic option for management of severe and extensive AD [36]. (ii) Topical formulation is a highly effective treatment for psoriasis of the face and flexures [39] and is proposed as an alternative treatment for inflammatory skin diseases in thin skin areas, as well as, pruritus ani [42]. In addition, it is effective in PG [51–53] and in cutaneous T cell lymphomas [60]. (iii) Topical formulation (0.1%) has been used for the management of OLP and may be effective and well tolerated in the treatment of anogenital lichen sclerosus [41]. (iv) Treatment of contact dermatitis is often palliative and directed against the cutaneous inflammation itself. (v) It has been shown to reduce the incidence of lupus dermatosis in the autoimmune-prone MRL/Mp-lpr/lpr (MRL/lpr) mouse. (vi) Better results in AA treatment are achieved in combination with intralesional steroids [50]. (vii) It can be considered both effective and safe for treating skin dermatosis in systemic lupus erythematosus (LE) patients [56].	(i) For severe active LE, its efficacy is considered limited at current dose settings and usage [55, 56]. (ii) There are contradictory results of tacrolimus as an inducer of skin cancer.

Sirolimus (rapamycin)	(i) A clinical trial has shown that macrolides, in a suitable formulation, can penetrate the human skin and exert beneficial effects for the treatment of psoriasis [75, 85].	(i) Contact sensitization to rapamycin could be developed [85].

Everolimus	(i) Potent immunosuppressive effects, antiproliferative properties, and anticancer effects have been observed. (ii) Effective in psoriasis treatment [87].	(i) It was ineffective in combination with prednisone or cyclosporine A in 2 patients with severe AD [88].

Temsirolimus	(i) Potent immunosuppressive effects, antiproliferative properties, and anticancer effects.	(i) Applications for different types of dermatitis are not yet known.

CSY0073	(i) Anti-inflammatory and immunomodulatory effects have been observed [93].	(i) Applications for different types of dermatitis are not yet known.

EM900 EM911	(i) Anti-inflammatory and immunomodulatory characteristics observed.	(i) Applications for different types of dermatitis are not yet known.

Ridaforolimus	(i) One of the first possible applications as an antitumor agent.	(i) Applications for different types of dermatitis are not yet known.
